# The Diagnostic Tampon: Its Value in the Recognition of Chronic Endometritis

**Published:** 1889-06

**Authors:** B. S. Schultze

**Affiliations:** Prof. of Gynecology, Director of the Lying-in Institution, and of the Gynecological Clinic, Jena; 2 Sellierstrasse


### *THE DIAGNOSTIC TAMPON: ITS VALUE IN THE RECOGNITION OF CHRONIC ENDOMETRITIS.*

BY DR. B. S. SCHULTZE,

Prof. of Gynecology, Director of the Lying-in Institution, and of the Gynecological Clinic, Jena.

IN No. 17 of the *Centralblatt für Gynäkologie*, 1880, page 393, I have mentioned a diagnostic agent that I have found of value during a number of years, which consists of a tampon of cotton (from which the fat has been removed), freely soaked in a 20 to 25 per cent. solution of tannin in glycerine, and firmly pressed in the vaginal vault, previously carefully cleaned, so that the mouth and vaginal portion of the cervix are completely covered. The glycerine in the tampon draws the water freely from the surrounding tissues, and permits the same to flow out, together with the water of the secretion. The formed constituents of the uterine secretion will not, or only to a slight degree, be floated over the place on which they come in contact with the tampon. If the tampon is removed after twenty-four or forty-eight hours, one finds on the same, in a wholly healthy uterus, corresponding to the place of the os uteri, only a small mass of cervical secretion, clear as glass. If the mucous membrane in a section above the mouth of the uterus is affected with catarrh, one finds, besides, on the tampon, pus which has come from the uterus during the same time. The tampon has attached itself so closely around the vaginal wall, that it takes off the most superficial epithelial layer of the vagina over its entire superficies.

I have used the tampon in this manner for twelve years, and can to-day confirm all that I said in 1880 concerning the diagnostic value of the same. I am not aware whether the diagnostic tampon has found a place here and there in practice, beyond the region of my personal agency and that of my pupils. In literature I have not found it spoken of, except a mention, unfavorable throughout, in Karl Schröder's *Handbook of Diseases of the Female Sexual Organs*, which mention has been repeated from edition to edition up to to-day.

If the question were only to find place for a diagnostic aid in a disease which, without the same, could be diagnosticated, I would gladly henceforth, as hitherto, leave opinions concerning the diagnostic tampon unconsidered, published now for the fourth time and with little effect. But it concerns the diagnosis of a very great number of cases of endometritis, in which, so far, as a matter of fact, the usual diagnostic means fail, and whose diagnosis must be in proportion to the indications placed at the disposal of physicians.

I said in this connection in the beginning of my before-mentioned article in 1880:

"The means generally recommended and used in the objective diagnosis of chronic endometritis are, in my opinion, not sufficient. They leave numerous cases unrecognized.

"As characteristic of chronic catarrh of the uterus, a more or less copious watery secretion is given in the newer handbooks. The diagnosis is to be made from this, if in slight affections of the cervix one sees an abundant watery secretion come out of the os uteri into the speculum. This sign does not occur in ten per cent. of the cases of uterine catarrh."

I said further:

"Neither the quantity nor the wateriness of the secretion is characteristic of endometritis, but the pus contained in it. The quantity of the secretion is so small in many cases, that the flow gives the woman no cause for complaint; indeed, it happens, when the cervix and vagina are not affected together, that, except in very marked cases, the secretion is so scanty that the patients, upon being questioned, make a bona fide assurance of not having a discharge."

My utterances naturally referred chiefly to the handbook of Karl Schröder, the most extensively used then and to-day in Germany, in which in the four editions (in print at that time) the symptoms of endometritis were treated in the same strain, *e.g.*:

"As in endometritis of the body the cervix also is concerned, we find likewise in the same the more pregnant symptoms which are present in cervical catarrh. In catarrh of the uterine cavity essentially the following occurs: In the first place, the watery discharge appears which, if in isolated cases is only of moderate intensity, not seldom becomes so profuse that it constitutes a copious blenorrhea, with proportionately clear discharge."

And the section "Diagnosis" begins with the sentence:

"If in the slighter affections of the cervix we see an abundant watery secretion come from the uterine mouth into the speculum, the whole uterine mucosa is the seat of disease. It is, indeed, doubtful whether we can recognize the especially affected parts always with certainty from the sensitiveness which they show to the sound."

In the sixth edition, my article on the Diagnostic Tampon receives mention, and in the following terms :

" The diagnostic tampon with which B. Schultze obtains the secretion of the cervix and uterine cavity, and from which, if pus is on it, he diagnosticates a purulent endometritis, is quite incapable of furnishing the proof that the pus comes from the cavity of the uterus. I consider, from my experience, a true purulent endometritis very rare, and I find that the diseased membrane in general secretes very little, but is inclined to hemorrhages in a high degree, not only at the menstrual periods, but also in the intervals. On this account, the hemorrhages, be they typical or atypical, are the most important symptoms of endometritis." (Sixth edition, page 120; Ninth edition, page 176.)

Then again, three pages farther on :

" The diagnostic tampon, so warmly recommended by B. Schultze, is wholly useless for an exact diagnosis, inasmuch as one cannot recognize on it what part of the secretion comes from the cervix, and what from the cavity of the uterus."

Before we weigh against one another the varying opinions concerning the diagnosis of a malady, and concerning the diagnostic value of an expedient, it is always advisable, primarily to be sure whether differences of meaning exist concerning the definition of the disease under consideration. Many a controversy over the diagnosis has resolved itself into this, that different things were understood under the same name, so that it was, therefore, not the diagnosis but the definition about which we were of different opinion.

The expression selected by Schröder, " If pus is on it, Schultze diagnosticates a purulent endometritis," makes the presumption strong that there is, perhaps, a difference of opinion with respect to the definition of the malady, so that from the pus resting thereon, Schröder would not diagnosticate a purulent endometritis. We formerly believed that we must keep catarrh and inflammation of the mucosa separate, but the following sentences immediately put aside this supposition. Schröder, as do we all, considers catarrh of the mucosa an inflammation. Simultaneously the following section bears as its title, in juxtaposition, and as meaning the same thing: Endometritis Cervicis, Catarrh of the Cervix; over this point, therefore, there is no difference of opinion, viz., that the mucous membrane, from which the pus comes that is found on the diagnostic tampon, is in a condition of inflammation.

The pus found on the tampon in front of the os uteri may come from the cervix, or the body of the uterus; it should also be considered whether it may not sometimes come from a tube. If one could recognize the source of the pus from its appearance, it would be, indeed, most agreeable in the matter of the diagnosis. But pus and pus look very similar, and only from accompanying circumstances can we form a judgment concerning its origin. Inasmuch as no part of the mucous membrane situated above the mouth of the uterus, neither that of the cervix, nor that of the body, nor that of the tubes, normally gives off pus, it is always, I think, of value to know that this membrane in some section is giving it off, and from attending conditions we can, in fact very often, determine from whence the pus comes. I said with regard to this:

"Very often the catarrhal affections of the cervix uteri are partly without, partly with, concurrent disease of the mucous membrane of the body of the uterus. In the outpoured secretion we recognized distinctly, as a rule, the origin of the same, whether from the cervix or the corpus uteri. The pus from the cervix is generally intimately mixed with the viscid, or gelatinous, cervical mucus. The pus from the corpus uteri does not show this mixing."

In a simultaneous affection of the mucous membrane of the neck and body, the purulent secretion of the cervix frequently preponderates in quantity so markedly, that a catarrh of the body existing next it cannot be recognized in the secretion, especially not when there is erosion or ectropion of the vaginal portion from the surface of which a copious thin pus, like that coming from the corpus uteri, is produced.

The cervical catarrh makes, as we know, very characteristic symptoms. It is most quickly and easily recognized with the speculum; so that in the cases in which the absence of a cervical catarrh is noted, the pus collected before the mouth of the uterus must be derived with tolerable surety from the corpus uteri. The easily perceived symptoms of endometritis cervicis are fully and excellently described in the older editions of Schröder's handbook. With regard to these symptoms, I proceed to the above quoted portion of my article on the Diagnostic Tampon:

"The above-mentioned change in the vaginal portion can, with perfect right, be considered as characteristic in catarrhal affections of the cervix: the erosions, the ectropion, and the swollen occluded follicles. If from the absence of this sign of cervical catarrh we could infer the absence of the latter, if the pus, unmixed with cervical mucus, and found upon the tampon in the wholly normal vaginal portion, can really come from the corpus uteri, catarrh confined to the body of the uterus is exceedingly frequent."

Therein is the essential difference between my opinion and that of Schröder, appearing in the last four editions of his handbook. Schröder says: "I consider, according to my experience, a true purulent endometritis to be very rare." On the contrary, I had shown how we could be convinced by collecting the pus, that endometritis is very common. Investigation for many years with the tampon has enlarged my knowledge in this direction, and it will do the same to every one who is not restrained by the remarks in Schröder's handbook from applying so simple a diagnostic expedient.

In the last four editions of the above book, at the above quoted place, we find further: "And I find that the diseased endometrium generally secretes very little." I find that also to be the case, but it was not Schröder's opinion when I wrote my article. And this little is purulent; and just because it is little, we do not observe it in the speculum without further procedure. But we must put before the os uteri an occluding tampon, in order to catch up the twenty-four hour secretion; and if we collect only a cubic millimeter of pus, it is pus nevertheless, and proves inflammation of the mucous membrane from whence it comes.

In opposition to the question of pus secretion from the diseased endometrium, spoken of in my article upon the diagnostic tampon, the handbook of Schröder renders the following prominent: "That the same is inclined to hemorrhages in a high degree, especially at the menstrual periods, but also in the intervals." And it further says: "On this account hemorrhages, be they typical or atypical, are the most important symptoms of endometritis."

The reader can accordingly come to the conclusion that the cases were similar, in which, according to Schröder the hemorrhages, and according to me the suppuration, was the diagnostic and most important symptom. Notwithstanding, as I stated above, that no difference exists between myself and Schröder's handbook concerning the definition of the term "endometritis," each of us here speaks, as a matter of fact, of different cases. It is without doubt, and undisputed, that irregular hemorrhages, be they typical or atypical, are the most important symptom of endometritis in all those cases in which this disease causes irregular hemorrhages; they are, for the woman who is attacked with them, the symptom most important and most injurious to her health. In the numerous cases in which endometritis causes profuse typical and atypical hemorrhages, it is also of importance still to ascertain that in the interval the diseased mucous membrane secretes some pus. *But whoever is acquainted with and acknowledges as such only those cases in which abnormal hemorrhages take place, fails to recognize the far greater number of cases of endometritis which do not, as yet, show these hemorrhages, or from their nature generally do not come to them, and these different forms and the early stages of endometritis can be recognized with the help of the diagnostic tampon.*

The section upon the diagnosis of endometritis in Schröder's handbook closes with the following sentences:

"In simple cases, therefore, the sound is sufficient to make the diagnosis; in the severer cases, however, there is no method freer from objections than the microscopical examination of a piece of mucous membrane. The diagnostic tampon so warmly recommended by B. Schultze—(then follows the above-quoted passage). I prefer the complete dilatation of the cervix, so that the finger can touch the whole uterine cavity only when I suspect polypi therein. In the diffuse forms of endometritis it has not the least advantage. Indeed, in such cases, the feeling communicated with the sound is surer than that with the finger."

The sound in the hand of the master is, undoubtedly, an admirable diagnostic instrument for recognizing the sensitiveness of the endometrium, as well as the consistence and unevenness of the mucous membrane that may exist, and also for examining as to the easy susceptibility to injury of the inner surface, and as to the inclination to hemorrhage. To the less experienced I cannot recommend the sound for the diagnostic inquiries under consideration, and the less expert ought to be able to recognize an endometritis. I am of the same opinion to-day concerning the questionable use of the sound as in 1880. I said, in the article on the Diagnostic Tampon:

"The sensitiveness of the uterine cavity to contact with the sound alleged by many as characteristic is, for two reasons, not to be depended upon. First, there are not a few cases of endometritis without sensitiveness of the cavity of the uterus to contact; and, secondly, sounding of the uterus is a diagnostic operation which often causes pain, if it is not performed by a wholly expert hand."

The much more usual consequences which arise when a person with a slightly trained hand makes the diagnostic attempt to cause pain with the sound, or to determine the consistence of the inner surface, I will not mention at all; for the present question it is sufficient that he misses the diagnosis.

We are indebted to the microscopical examination of the diseased uterine mucosa, scraped off from the living woman, for the very marked advances in our knowledge of its pathology; the probability offered to the clinical investigator and teacher by this method is not great enough for him to place side by side the clinical picture and the anatomical condition, taken from the same case. Whether the method is of equal value for the diagnosis of individual cases, must already, therefore, come into question, because it requires a practice in the use of the microscope, and an experience in judging the microscopical appearances met with, which cannot everywhere be presupposed; moreover, to consult the pathological anatomist concerning every suspected case, is not practicable. But even in the hand of the experienced microscopist this method is not wholly free from objections; in the first place, because the endometritis, in many cases, is limited to one side of the uterus, or to one portion of the same; the removed portion can, therefore, easily be healthy mucous membrane, while endometritis exists in other places. In the second place, because even the piece of mucosa, directly under consideration, does not always suggest something pathological. Küstner says in this connection:[1]

"It is among the impossibilities to differentiate in the bare-scraped pieces the most frequent form of endometritis hyperplastica from a normal uterine mucosa, shortly before menstruation."

The proposition concerning the palpation of the whole cavity of the uterus refers to the recommendation, repeated many times by me, of this diagnostic procedure.[2]

The palpation of the cavum uteri to the fundus and the necks of the tubes with the finger, after previous dilatation, has been warmly commended by me for the diagnosis of the condition of the inner surface, especially for the recognition of limited exuberant mucous membrane, of polypi, decidual remains, carcinoma of the uterine body—in short, for all cases suspected ot having one of the above-mentioned diseases, after I have divested the procedure of all danger by the method devised by me of antiseptic dilatation, and through copious irrigation with carbolic acid solution. I use the just-mentioned diagnostic procedure in many cases in which others simply scrape out the uterus, and I have, by touching the uterine cavity with the finger, learned the cause of long-existing hemorrhage, and thus succeeded in obtaining the indication for its removal in cases where others—renowned medical men, too—had curetted the uterus repeatedly and fruitlessly. I have, for this reason, given the opinion that, where atypical hemorrhage exists, independent of some sudden

1. Otto Küstner—Beiträge zur Lehre von der Endometritis, Jena, 1883, S. 17.

2. Die Erweiterung des Uterus durch Laminaria digitata.—*Centralbl. f. Gyn.*, 1878, Nr. 7. Ueber Indication und Methode der Dilatation des Uterus.—*Wiener Med. Blätter*, 1879, Nr. 42-45. Zur Dilatation des Uterus.—*Archiv f. Gyn.*, xx., 1882.

procedure adopted for stopping the flow, I consider it entirely indicated to palpate the cavum uteri with the finger before we determine upon further treatment.

In the essay on the Diagnostic Tampon, I spoke, *en passant*, of the digital examination of the body of the uterus, and in the following words:

"The digital palpation of the cavum uteri, though very often indicated because of endometritis, cannot always be used as a diagnostic means, for the reason that those cases which afford tangible results are the minority."

I do not know whether it is this sentence or some other to which the remark in the last paragraph of the section on the treatment of endometritis in Schröder's handbook refers. The same says[3]:

"In more recent times, the irrigation of the uterine mucosa and the curetting of the same has appeared in the foreground in the treatment of endometritis. Most of the later gynecologists proceed in a way similar to that which I have above represented. B. Schultze attaches especial value to dilatation of the uterine cavity of such a kind that the finger can touch the same. In cases of polypi, with an otherwise normal mucous membrane, his procedure of complete dilatation and nipping off of the polypi is rational. But polypi of the mucous membrane of the uterus and chronic endometritis are essentially different things. On this account, Schultze's procedure can raise no claim to be a treatment of chronic endometritis."

Most certainly not. Auscultation of the thorax can make no pretence to be treatment of chronic pneumonia. The illumination of the fundus of the eye is no remedy for nephritis, and the diagnostic touching of a broken limb is no plaster-of-paris bandage.

I will now devote a brief consideration to some important features (features already mentioned in my article of 1880) of those cases of endometritis which, neither through profuse bleeding nor through copious watery secretion, direct the attention of the patient and of the physician to the genital malady, and for the recognizing of which the diagnostic tampon is of value on this account.

---

3. Handbuch der Krankheiten der weiblichen Geschlechtsorgane, 6. Auflage, 1884, S. 129. 9. Auflage. Umgearbeitet und herausgegeben von M. Hofmeier, 1889, S. 186.

Many cases of dysmenorrhea and sterility,—that dysmenorrhea which manifests itself in pain one or more days previous to the flow, and that sterility in which, in the first years at least, careful palpation shows no departure from the normal condition,—depend upon the form of endometritis under consideration. The dysmenorrhea passes for "nervous,"—an expression, by the way, against which no objection exists, for every pain is nervous,—narcotics and other nerve remedies, likewise salicylic acid and warm poultices, at times help this pain; but where the same exists for years, palliative remedies fail.

When virgins afflicted with dysmenorrhea, and sterile wives, finally go to the gynecologist, there have been already added to the endometritis complications, such as parametritis, changes in the position of the uterus, and oöphoritis, which, through the symptoms proceeding directly from them, stand in the foreground, and the preëminence of which appears in the matter of the indications. It is important not to overlook the accompanying endometritis, lying, in my opinion, at the root of the whole disease, and, at the conclusion of the special cure, to examine as to the pus secretion from the uterus, and to also free the patient, if she desires, from her endometritis. The chief advantage from this will be, that she is far safer from relapses of her disease.

I will also state, at this opportunity, that endometritis, running its course without irregular bleeding and without profuse secretion, occurs, which is so inveterate and obstinate that only a thorough curetting of the uterus cures it.

If girls with dysmenorrhea and barren women come exceptionally early under gynecological observation, at a time when accurate palpation and examination with the speculum reveals no deviation from the normal, we should use the diagnostic tampon before we either declare baths the most suitable remedy, or, on the other hand, immediately proceed to bilateral section of the cervix. In very many cases it will show endometritis; and if, corresponding to the diagnosis, after previous laminaria dilatation when necessary, we methodically irrigate, the dysmenorrhea ceases. Then, after the expiration of one or two menstrual periods, if we use the diagnostic tampon, we find that no more pus comes from the uterus. After similar treatment the women formerly sterile conceive, it being supposed that, as for the rest, the conditions requisite thereto are present. As I have seen the above-noted therapeutic results appear in many cases during the course of years, I consider myself entitled to pronounce endometritis, manifesting itself through suppuration so slight as to be scarcely otherwise recognized than with the diagnostic tampon, to be a frequent cause of dysmenorrhea and sterility.

A great number of symptoms designated as nervous and as hysterical,—foremost, nervous dyspepsia, then lumbar pain, migraine, asthmatic attacks, and nervous cough,—are sometimes dependent upon the same endometritis, accompanied with slight suppuration. I obtained the evidence of this, anew, from the consequences of corresponding treatment. The number of patients is very great who have been treated for years for stomach trouble, who have also been declared sound in the genital organs by gynecologists whom they have occasionally consulted, because palpation and the speculum gave nothing abnormal, and who, after the diagnostic tampon demonstrated to me purulent endometritis, were freed from their difficulty by means of dilatation and methodical irrigation of the uterus. The results of treatment relative to the individual symptoms,—for example, gastralgia, lumbar pain, headache, convulsive cough,—are often most brilliant, so that, upon the first thorough carbolic or sublimated injection, disorders which have appeared daily for years, disappear and, with continued methodical treatment, stay permanently away. Even severe hysterical symptoms have not seldom, *ex juvantibus*, appeared to me dependent upon endometritis, when accompanied with the little suppuration already spoken of.

It is not the material loss, through suppuration, that so often reduces patients in the above-mentioned category to such a marked degree; an only moderately well-nourished body can bear, without harm, for years the waste from a few cubic millimeters of pus daily; often there is not more. It is the effect upon the nervous system, occasioned by the situation of the affection, that gives the endometritis spoken of this great significance for the general health.

In this influence (upon the general health), the stagnation of the secretion seems to play an essential role. Those uterine catarrhs (they are the more rare), whose profuse serous secretion sweeps out the pus in a free discharge, do not, according to my observation, long injure the general health to any degree even when attended with much greater loss of material. The conditions in the uterine mucous membrane are essentially more favorable for stagnation than in other mucous membranes, because there is no secretion or excretion produced near by to wash out the pus. If through endometritis swelling of the mucous membrane about the orificium internum supervenes, the scanty pus stagnates the more completely.

It may be remarked, by the way, that the fact that the secretion at times stagnates, and that, therefore, pus does not escape from the uterus during every day that the endometritis exists, is important from a diagnostic point of view; absence of pus, once, on the diagnostic tampon does not exclude with certainty the existence of purulent endometritis.

Not only the discharge of pus, but its secretion as well, is periodic in many cases. The so-called interval pain (mittelschmerz) is, as I have already said in that article, frequently accompanied with pus secretion, limited to a few days, or even only hours, in the middle of the interval between two periods. Observation with the tampon demonstrates this.

It appears that stagnation of the pus favors parametritis, mostly chronic from the beginning, which is very often added to the chronic purulent endometritis after long duration of the same. In married women and virgins, who come under treatment with chronic parametritis, the tampon almost always shows purulent secretion (also where cervical catarrh does not exist), and the history is then almost invariably, that a slight flow, which was not taken notice of, and dysmenorrheal difficulty, which passed for nervous, had long preceded the appearance of the parametritis. Chronic parametritis, most especially the parametritis posterior, leads either to shortening of one of Douglas' folds, and therewith to torsion and to pathological anteflexion, or to fixation of the cervix more in front or to complete relaxation of Douglas' folds, and with it, in the two latter cases, to retroflexion of the uterus; in the further course perimetritis and oöphoritis finally appear.

I consider, from my observations and after careful taking of numerous histories, chronic endometritis,—particularly that form which finds its expression neither in profuse secretion nor in irregular hemorrhages,—to be the terminal process of many, perhaps of the most, of the inflammatory occurrences running a chronic course from the beginning in the para- and perimetrium. That primary uterine catarrh often leads to metritis and parametritis, is beyond doubt, and it is just as much beyond doubt that retroflexion of the uterus often follows parametritis posterior that has ceased. Detailed remarks on this subject are contained in my essays in Vols. IV. and VIII. of the Archiv für Gynäkologie[1].

Through retroversion and retroflexion of the uterus, the ovaries necessarily suffer a change in position backwards, on account of which they become removed from their normal protected position under the ala vespertilionis, and exposed to manifold insults[2].

In consequence of the changed position occasioned by retroflexio uteri, the ovaries swell and become painful, demonstrable from the fact that after successful reposition of the uterus they recover their normal position, and the swelling, for the most part, soon disappears. But not only inflammatory processes in the ovaries are produced by retroflexion of the uterus: it is more than probable that a great number of ovarian tumors can thank their origin to the position conditioned by the long-

1. Ueber Versionen und Flexionen, speciell über die mechanische Behandlung der Rückwärtslagerungen der Gebärmutter. Archiv f. Gynäk., IV., 1872, S. 373. Ueber die pathologische Anteflexion der Gebärmutter und die Parametritis posterior. Archiv f. Gynäk., VIII., 1875, S. 134.

2. Zur Kenntniss der Lage der Eingeweide im weiblichen Becken. Archiv f. Gynäk., IX., 1876, S. 262.

existing retroflexion of the uterus. To give the arguments for this, would lead us too wide from the subject. For those who wish to know more of this highly interesting question, I quote the places in which I have considered the same[1].

If my opinion on the etiological significance of simple uterine catarrh is right regarding the many greater and serious diseases of the genital organs, it is of so much the greater importance to recognize these uterine catarrhs in time, in order to derive from this recognition the corresponding indications.

I believe that a tolerably recent uterine catarrh, by judicious holding in check, and especially by averting further injurious agencies, even if not so light as catarrhal disease of other mucous membranes, can recover without direct treatment of the diseased membrane.

In as much as I have proved, so far as is possible without bacteriological investigation, that uterine catarrhs develop mostly from atmospheric infection[2], I understand the warding off of further injurious agencies to be essentially the averting of further atmospheric infection.

In the well-closing virgin vulva, opportunity for infection from the atmosphere occurs only at the menstrual period through the moist track of the outflowing blood. If catarrh already exists, the opportunity continues on account of the secretion moistening the outer parts, and when the vulva gapes,—for instance, from a perineal rupture of even quite limited extent,—the atmosphere with its germs of infection has a wholly free entrance into the vagina.

To the conditions for the cure and for the prophylaxis belongs, on this account, as most essential, the greatest cleanliness of the outer genitalia at the menstrual period and during the existence of any other flow whatsoever, and the keeping away of the air from the same at this time. To this end I have recommended, for all girls and women, antiseptic bandaging of the

1. Die Pathologie und Therapie der Lageveränderungen der Gebärmutter. Berlin, 1881, §§ 56 u. 130.

2. Zur Aetiologie and Prophylaxe der Genitalerkrankungen des Weibes.—*Wiener Med. Blätter*, 1882, No. 12.

genitalia at the time of the menses, from the first appearance of the same on, and I rejoice that my proposal finds favor in ever-widening circles, and a constantly more general application by means of the many antiseptic menstruation bandages recommended by the makers.

Where catarrh already exists, the atmosphere must be kept away by means of appropriate clothing and corresponding layers of antiseptic cotton at other than the menstrual periods; and also small perineal ruptures, in so far as they detract from the ability of the vulva to keep shut, must be closed by operation.[1]

As stated, the women affected with endometritis come for the most part late under the observation of the gynecologist, when either the disease of the mucous membrane has gone on so far that it gives occasion to irregular hemorrhages, or when, even without their existence, inflammatory affections of the pelvic organs, or changes in position of the uterus, have appeared along with it, and which, through the preponderating significance of their symptoms, conceal the uterine catarrh which is, perhaps, the cause of the same.

The earlier stages of chronic endometritis are, as I have already made prominent in the first article on the diagnostic tampon, mostly unrecognized in non-gynecological practice. The anemia, the chlorosis, the gastralgia, the other dyspeptic symptoms, the pain in the lumbar region, the migraine, the hysterical convulsions, perhaps also the dysmenorrhea, are objects of more or, for the most part, less successful treatment, till finally, in the above-designated way, the genital malady appears in the foreground. Often years have gone by with fruitless treatment, and the patients have been markedly reduced in their whole nutrition; the treatment is now one of greater detail, and for the patient more burdensome and lengthy. In the earlier stages, if the diagnosis of local disease had been made, perhaps not even local treatment would have been successful. Through appropriate diet, and through judiciously selected baths and springs, perhaps the catarrh would run out its course without

1. Pathologie und Therapie der Lageveränderungen, §§ 59 u. 61.

leading to complications; or, if a corresponding local treatment had been earlier employed, the same would have led to cure much sooner, and with greater surety from relapses.

One cannot determine so easily to consult the gynecologist in the early stages of a chronic endometritis, where the suspicion is far from the patient and the physician, to refer the stomach difficulty, or the migraine, to the genital malady. It is likewise not pleasant for the physician to have occasioned a consultation with the specialist, and to receive the information that the organs concerned are wholly sound. On this account, a diagnostic agent, which gives answer with equal exactness to the practitioner as to the specialist, is especially to be prized in practice.

Gynecological local therapy cannot well be the common property of all the much-occupied physicians in the whole domain of medicine. Gynecological diagnosis should be such in its widest range. From exact gynecological diagnosis there proceed other very numerous and very important indications like those in therapeutics. Whether an anemia, whether a chlorosis, whether a migraine, or a dyspepsia, is complicated with the genital malady, is at any rate of value to know. Whether the latter is to be conceived as the cause, forms a further important question, and this again a further one, whether local treatment is indicated. The answer to these questions may show one thing or another; at any rate, the knowledge of these complications to the circumspect physician will influence the placing of the medication to the advantage of the patient, and will sharpen further observation.

Inasmuch as uterine catarrh is one of the commonest diseases of the female genital organs, one of those which, more than many others, calls forth in the way of reflexes in distant organs symptoms of disease which easily yield to another interpretation; inasmuch as the significance of simple purulent uterine catarrh is a great one for the entire organism, firstly on account of the sterility which it occasions, then because it reduces the patient through the nervous symptoms; finally, and not least, for the reason that it is the initial disease of far more severe ones of the genital apparatus—it is important that its diagnosis should not escape the physician, and therefore it is of especial value to possess a diagnostic expedient whose application does not even require special gynecological knowledge. This we have in the diagnostic tampon.

2 SELLIERSTRASSE, JENA, April 30, 1889.

